# Chemical Composition, Antifungal and Antibiofilm Activities of the Essential Oil of *Mentha piperita* L.

**DOI:** 10.5402/2012/718645

**Published:** 2012-12-13

**Authors:** Mohammad Jamal Saharkhiz, Marjan Motamedi, Kamiar Zomorodian, Keyvan Pakshir, Ramin Miri, Kimia Hemyari

**Affiliations:** ^1^Department of Horticultural Sciences, Faculty of Agriculture, Shiraz University, Shiraz 71441-65186, Iran; ^2^Department of Medical Mycology and Parasitology, School of Medicine, Shiraz University of Medical Sciences, Shiraz 71348-45794, Iran; ^3^Center of Basic Sciences in Infectious Diseases, School of Medicine, Shiraz University of Medical Sciences, Shiraz 71348-45794, Iran; ^4^Medicinal and Natural Products Chemistry Research Center, Shiraz University of Medical Sciences, Shiraz, Iran

## Abstract

Variations in quantity and quality of essential oil (EO) from the aerial parts of cultivated *Mentha piperita* were determined. The EO of air-dried sample was obtained by a hydrodistillation method and analyzed by a gas chromatography/mass spectrometry (GC/MS). The antifungal activity of the EO was investigated by broth microdilution methods as recommended by Clinical and Laboratory Standards Institute. A biofilm formation inhibition was measured by using an XTT reduction assay. Menthol (53.28%) was the major compound of the EO followed by Menthyl acetate (15.1%) and Menthofuran (11.18%). The EO exhibited strong antifungal activities against the examined fungi at concentrations ranging from 0.12 to 8.0 **μ**L/mL. In addition, the EO inhibited the biofilm formation of *Candida albicans* and *C. dubliniensis* at concentrations up to 2 **μ**L/mL. Considering the wide range of the antifungal activities of the examined EO, it might be potentially used in the management of fungal infections or in the extension of the shelf life of food products.

## 1. Introduction

Medicinal plants have been used for centuries in traditional medicine because of their therapeutic value. Mint species have been exploited by man for more than two thousand years. Peppermint itself has been used for more than 250 years [1].* Mentha piperita, *(family Lamiaceae) is a species found in Iran and many parts of the world which has an economical value for its flavoring, odor, and therapeutic properties in foods and cosmetic industrial products. In addition, the leaves and flowers of *M. piperita* have medicinal properties [2, 3]. Essential oils are valuable natural products used as raw materials in many fields including perfumes, cosmetics, aromatherapy, phototherapy, spices, and nutrition. Peppermint (*M. piperita*) oil is one of the most popular and widely used essential oils, mostly because of its main components, Menthol, and menthone [4]. Previous studies have shown antiviral [5], antibacterial [6, 7], antifungal [6, 8–10], antibiofilm formation [11–13], radioprotective [14], antioedema [15], analgesic [16], and antioxidant activities [6] of the EO and methanolic extracts of herbal parts and callus cultures of *M. piperita*. In addition, *M. piperita *EO has been shown to cause inhibitory effects against radial fungal growth and aflatoxin production by *Aspergillus* species [17].

In the past two decades, the emergence of resistance to various antifungal drugs has accelerated dramatically. Azole-resistant *Candida* and *Aspergillus* species are the top pathogens responsible for nosocomial or food-borne infections [18, 19]. In addition, the formation of biofilms by *Candida* species have raised concerns due to their increased resistance to antifungal therapy and protects the microbial cells within biofilms from the host immune defenses [20–23]. 

An alternative approach to overcome antibiotic resistance might be using natural products and phytochemicals. It has also been shown that some plant extracts efficiently inhibit the biofilm formation of *C. albicans *[24]. Moreover, EOs especially with known antibacterial effects have the potential to be used in food industry as preservatives and to increase the shelf life of products. Therefore, determining the antimicrobial properties of EOs might help to overcome microorganism resistance to antibiotics and prevent food spoilage. The chemical composition of aromatic plants depends largely on the individual genetic variability and different plant parts [25–27]. The presence and concentration of certain chemical constituents of EOs also fluctuate according to the season, climatic condition, and site of plant growth [25, 26].

The goal of this study was to investigate the chemical composition and *in vitro* antifungal and antibiofilm activities of essential oils of the leaves of *M. piperita *collected in the region of Fars from Iran.

## 2. Methods and Material 

### 2.1. EO Preparation

At full flowering stage, the aerial parts of the *M. piperita* were hydrodistillated for 2.5 h, using an all-glass Clevenger-type apparatus, according to the method outlined by the British Pharmacopoeia. The sample oils were dried over anhydrous sodium sulfate and stored in sealed vials at 4°C before gas chromatography and gas chromatography-mass spectrometry (GC-MS) analysis. 

### 2.2. EO Analysis by Gas Chromatography-Mass Spectrometry

The EO was analyzed by GC-MS. The analysis was carried out on a Thermoquest-Finnigan Trace GC-MS instrument equipped with a DB-5 fused silica column (60 m × 0.25 mm i.d., film thickness 0.25 mm). The oven temperature was programmed to increase from 60°C to 250°C at a rate of 4°C/min and finally held for 10 min; transfer line temperature was 250°C. Helium was used as the carrier gas at a flow rate of 1.1 mL/min with a split ratio equal to 1/50. The quadrupole mass spectrometer was scanned over the 35–465 amu with an ionizing voltage of 70 eV and an ionization current of 150 mA.

GC-flame ionization detector (FID) analysis of the oil was conducted using a Thermoquest-Finnigan instrument equipped with a DB-5 fused silica column (60 m ×  0.25 mm i.d., film thickness 0.25 mm). Nitrogen was used as the carrier gas at the constant flow of 1.1 mL/min; the split ratio was the same as that for GC-MS. The oven temperature was raised from 60°C to 250°C at a rate of 4°C/min and held for 10 min. The injector and detector (FID) temperatures were kept at 250°C and 280°C, respectively. Semiquantitative data were obtained from FID area percentages without the use of correction factors.

### 2.3. Identification of EO Components

Retention indexes (RIs) were calculated by using retention times of *n*-alkanes (C6–C24) that were injected after the oil at the same temperature and conditions. The compounds were identified by comparing their RI with those reported in the literature, and their mass spectrum was compared with those reported in Wiley Library [28].

### 2.4. Determination of Antimicrobial Activities

#### 2.4.1. Microorganisms

The antifungal activities of the EO against 25 standard strains of fungi, including *C. albicans* (ATCC 5982, 1912, 562, 1905, 1949, 10261, and 2730),* C. tropicalis* (ATCC 750), *C. krusei* (ATCC 6258), *C. glabrata* (ATCC 863, 2192, 2175, 6144, and 90030),* C. dubliniensis* (CBS 8501, ATCC 8500, 7987, and 7988), *C. parapsilosis *(ATCC 4344),* C. neoformance *(ATCC 9011),* Aspergillus flavus* (ATCC 64025), *A. fumigatus* (ATCC 14110, CBS 144.89),* A. clavatus* (CBS 514.65),* and A. oryzae* (CBS 818.72) were determined. In addition, the antifungal activities of the EO against 35 clinical isolates of yeasts identified by polymerase chain reaction-restriction fragment length polymorphism (PCR-RFLP) were also examined [29, 30]. The antifungal susceptibility of clinical isolates of the tested fungi against fluconazole was examined by microdilution and disk diffusion methods [31, 32]. 

#### 2.4.2. Determination of Minimum Inhibitory Concentration

Minimal inhibitory concentrations of the EO against standard and clinical species of the fungi were determined by the broth microdilution method as recommended by the Clinical and Laboratory Standards Institute (CLSI), with some modifications [31, 32]. Briefly, the RPMI-1640 (with L-glutamine and phenol red, without bicarbonate) (Sigma, USA) was prepared and buffered at pH 7.0 with 0.165 mol 3-(N-morpholino)propane sulfonic acid (MOPS) (Sigma-Aldrich, Steinheim, Germany). Serial dilutions of the EO (0.06 to 16 *μ*L/mL) were prepared in 96-well microtiter trays using RPMI-1640 media (Sigma, St. Louis, MO, USA) buffered with MOPS (Sigma, St. Louis, MO, USA). Double dilutions of Fluconazole were also prepared for each of the tested fungi with the final concentration of 0.25 to 128 *μ*g/mL. Stock inoculums were prepared by suspending three colonies of the examined yeast in 5 mL sterile 0.85% NaCl and adjusting the turbidity of the inoculums to 0.5 McFarland standard at 630 nm wavelength (this yields stock suspension of 1–5 × 10^6^ cells/mL). For moulds (*Aspergillus* spp.), conidia were recovered from the 7-day-old cultures grown on potato dextrose agar by a wetting loop with Tween 20. The collected conidia were transferred in sterile saline and their turbidity was adjusted to optical density of 0.09 to 0.11 that yields 0.4–5 × 10^6^ conidia/mL. Working suspension was prepared by making a 1/50 and 1/1000 dilution with RPMI of the stock suspension for moulds and yeasts, respectively. After the addition of 0.1 mL of the inoculums to the wells, the trays were incubated at 30°C for 24–48 h in a humid atmosphere. 200 *μ*L of the uninoculated medium was included as a sterility control (blank). In addition, growth controls (medium with inoculums and 5% (v/v) without the EO or fluconazole) were also included. The growth in each well was compared with that of the growth control well. MICs were visually determined and defined as the lowest concentration of the EO that produced no visible growth. Each experiment was performed in triplicate. In addition, minimum fungicidal concentrations (MFCs) of all the examined agents were also determined by culturing 10 *μ*L from the wells showing no visible growth onto SDA plates. MFCs were of the lowest concentration that showed either no growth or fewer than 4 colonies, which corresponded to 98% killing activity of the initial inoculums. MFCs were determined as the lowest concentration yielding no more than 4 colonies, which corresponds to a mortality of 98% of the fungi in the initial inoculums.

### 2.5. Inhibition of Biofilm Formation

#### 2.5.1. Biofilm Formation and Growth

According to Behnam et al. [10], *C. albicans *and* C. dubliniensis *strains were maintained on Sabouraud dextrose agar (Difco) plates. After 48 hrs one loop of the colonies was transferred to 20 mL Sabouraud broth in 250 mL Erlenmeyer flasks and incubated overnight in an orbital shaker (100 rpm) at 30°C under aerobic condition. Yeast cells were harvested and washed twice in sterile phosphate-buffered saline (PBS) (0.8% [w/v], NaCl (Merck); 0.02% [w/v], KH_2_PO_4_ (Merck); 0.31% [w/v], Na_2_HPO_4_+12H_2_O (Merck); 0.02% [w/v], KCl (Panreac); pH 7.4). Then, they were resuspended in RPMI 1640 supplemented with L-glutamine (Gibco) and buffered with morpholinopropanesulfonic acid (MOPS) and the cell densities were adjusted to 1.0 × 10^6^ cells/mL after counting with a hemocytometer. Serial dilution of the EOs (0.015–8 *μ*L/mL) in RPMI 1640 was prepared in a presterilized, polystyrene, flat-bottom, 96-well microtiter plates (Nunc). After the addition of 0.1 mL of the yeast inoculums to the wells, the trays were incubated at 30°C for 24–48 h in a humid atmosphere. 200 *μ*L of the uninoculated medium was included as a negative control (blank). In addition, RPMI with yeasts but without the EOs served as positive controls. 

#### 2.5.2. Biofilm Inhibition Assay

A semiquantitative measure of biofilm formation was assayed using a 2, 3-bis(2-methoxy-4-nitro-5-sulfo-phenyl)-2H-tetrazolium-5-carbox-anilide (XTT) reduction assay. XTT (Sigma Chemical Co.) was prepared as a saturated solution at a concentration of 0.5 mg/mL in Ringer's lactate. This solution was filter-sterilized through a 0.22 *μ*m-pore-size filter, divided into aliquots and then stored at −70°C. Prior to each assay, an aliquot of the XTT stock solution was thawed and treated with menadione sodium bisulfite (10 mM prepared in Distilled Water; Sigma Chemical Co.) to obtain a final concentration of 1 *μ*M of menadione. A 100 *μ*L aliquot of XTT menadione was then added to each prewashed wells. The plates were then incubated in dark for 2 hours at 37°C, and the colorimetric change at 490 nm (a reflection of the metabolic activity of the biofilm) was measured with a microtiter plate reader (Titertekplus-MS2 reader, UK) [10].

## 3. Results and Discussion

### 3.1. Results

Approximately 17 compounds, representing 99.37% area of the oil, were identified. The qualitative and quantitative compositions of the EO of *M. piperita* are presented in Table 1. GC/MS analyses showed that the main constituent of the EO was Menthol (53.28%) followed by Menthyl acetate (15.1%) and Menthofuran (11.18%).

The antifungal activities of *M. piperita* EO against the tested yeasts are shown in Table 2. The EO inhibited the growth of all of the tested yeasts at concentrations of 0.12–4 *μ*L/mL. Furthermore, the EO exhibited fungicidal activity (MFC) for all of the above-mentioned yeasts at concentrations ranging from 1 to 8 *μ*L/mL. No significant differences in inhibitory concentrations were found between azole-resistant and -susceptible strains. In addition, the EO inhibited the growth and killed the standard strain of *Cryptococcus neoformance* at the concentration of 4 *μ*L/mL. All of the *Aspergillus* standard strains were susceptible to *M. piperita* EO at concentrations of 0.5–4 *μ*L/mL (Table 3). As shown in Table 4, the EO completely inhibited the biofilm formation of *C. albicans* and *C. dubliniensis* at concentrations of 1 *μ*L/mL and 2 *μ*L/mL, respectively. 

### 3.2. Discussion

The compositions of the EOs might be affected by the developmental stages of the plant. Although some authors reported alpha terpinen as the dominant component of *M. piperita* EO (19.7%), similar to the previous studies [4, 7, 33], we identified Menthol as one of the main constituents of the EOs. The higher concentration of Menthol in this study as compared to some of those of previous reports [4, 34] may reflect variations due to geographical location from which the plants were collected. 

In this study, the EOs exhibited fungistatic and fungicidal activities against both of the standard and clinical strains of *Candida* species at concentrations ranging from 0.5 *μ*L/mL to 8 *μ*L/mL, which can be best compared to the previous investigations [6, 8, 9, 33]. One of the encapsulated yeasts, *C. neoformance,* is a well-known primarily opportunistic pathogen which produces chronic and life-threatening meningitis. According to the findings of this study, the examined oils killed the standard strain of *C. neoformance* at concentration of 4 *μ*L/mL. Similar to the previous studies [10, 17] the EO of *M. piperita* exhibited a strong anti-*Aspergillus* activity with MIC values ranging from 0.5 to 4 *μ*L/mL. MFCs of the EO against the tested fungi were almost similar or two times greater than those of their corresponding MICs. 

Since the EOs exhibited similar antifungal effect against the tested azole-resistant and azole-susceptible strains, it could be assumed that the mechanism of the action of the EOs is different with those of the above-mentioned antifungal drug. One of the main characteristics of EOs is their hydrophobicity, which enables their incorporation into the cell membrane. The tested EO was rich in Menthol. It has been shown that this phenolic monoterpene has a hydroxyl group around the phenolic ring and exhibits its antimicrobial activity through the disruption of the cytoplasmic membrane [35, 36]. Biofilm formation by *Candida* species is a phenomenon which helps the survival, pathogenesis, and drug resistance. The most commonly *Candida* species associated with biofilm formation is* C. albicans*. In the present study, the formation of biofilm was inhibited completely at a concentration of up to 2 *μ*L/mL in a dose-dependent manner, which was comparable to the study of Agarwal et al. [11]. 

## 4. Conclusion 

As the industries tend to reduce the use of chemical preservatives in their products, EO of *M. piperita* with potential active antimicrobial properties might be considered as a natural source for the maintenance or extension of the shelf life of products. In addition, delectable taste of the EO at the concentrations needed for antimicrobial properties was a bonus to its antimicrobial effects. On the other hand, these EOs might also be considered for developing products for controlling fungal infections. As these tests have all been done *in vitro*, the next step may be further investigations in animal models to see if infection can be inhibited by the EO.

## Figures and Tables

**Table 1:** Chemical components (%) of the essential oils distilled from *Mentha piperita*.

*Mentha piperita* essential oil		
Compound	RI^a^	% in oil
*α*-Pinene	939	0.32
Sabinene	975	0.26
*β*-pinene	979	0.58
1,8 Cineole	1031	6.69
*cis*-Sabinene hydrate	1070	0.50
Menthone	1152	2.45
Menthofuran	1164	11.18
Neomenthol	1165	2.79
Menthol	1171	53.28
Neomenthyl acetate	1273	0.65
Menthyl acetate	1295	15.10
Isomenthyl acetate	1305	0.61
*β*-Bourbonene	1388	0.37
(z)-Caryophyllene	1408	2.06
E-*β*-farnesene	1456	0.30
Germacrene D	1485	2.01
Bicyclogermacrene	1500	0.22

Total		99.37

^a^Retention indices on DB-5 column.

**Table 2:** Antifungal activity (MIC and MBC) of the essential oil of *Mentha piperita* against standard and clinical isolates of yeasts.

		*Mentha piperita* essential oil	
	Strain (number of isolate)	MIC (*μ*L/mL)	MFC (*μ*L/mL)
		GM^a^ (range)	GM^a^ (range)
Standard isolate	*C*. *albicans *(6)	1.5 (1-2)	3.3 (2–4)
	*C*. *glabrata *(5)	1.2 (0.5–2)	2.8 (2–4)
	*C*. *tropicalis *(1)	1.0	2.0
	*C*. *krusei *(1)	0.5	1.0
	*C*. *dubliniensis *(5)	2.4 (2–4)	6.0 (4–8)
	*C*. *parapsilosis *(1)	4.0	4.0
	*C*. *neoformance *(1)	4.0	4.0

Azole-sensitive clinical isolates	*C*. *albicans *(9)	2.0 (0.12–4)	2.8 (2–4)
	*C*. *dubliniensis *(3)	0.6 (0.5–1)	1.6 (1-2)
	*C*. *tropicalis *(2)	2.0	2.0
	*C*. *parapsilosis *(3)	1.1 (0.5–2)	4.0
	*C*. *glabrata *(1)	0.12	4.0

Azole-resistant clinical isolates	*C*. *albicans *(12)	1.6 (1-2)	2.3 (1–4)
	*C*. *dubliniensis *(2)	0.5	2.5 (1–4)
	*C*. *tropicalis *(3)	3.0 (2–4)	3.0 (2–4)

^a^Geometric mean; MIC: minimum inhibitory concentration; MFC: minimum fungicidal concentration.

**Table 3:** Antifungal activity (MIC and MFC) of the essential oil of *Mentha piperita* against standards *Aspergillus* species.

Strain	*Mentha piperita* essential oil	
	MIC (*μ*L/mL)	MFC (*μ*L/mL)
*A. flavus* (ATCC 64025)	4.0	8.0
*A. fumigatus* (ATCC 14110)	0.5	2.0
*A. fumigates* (CBS 144.89)	2.0	8.0
*A. oryzae* (CBS 818.72)	2.0	4.0
*A. clavatus *(CBS 514.65)	0.5	1.0

**Table 4:** Antibiofilm activity of the essential oil of *Mentha piperita* against biofilm formation caused by *Candida albicans* and *Candida dubliniensis*.

*Mentha piperita *	*C. albicans *		*C. dubliniensis *	
Essential oil concentration *μ*L/mL	Optical density	% viability	Optical density	% viability
8	0.036	0.0	0.036	0.0
4	0.036	0.0	0.037	0.0
2	0.038	0.0	0.041	0.0
1	0.039	0.0	0.102	23.2
0.5	0.297	40.0	0.173	51.5
0.25	0.311	43.1	0.173	51.5
0.12	0.337	47.3	0.178	53.4
0.06	0.394	56.3	0.191	58.2
0.03	0.416	59.8	0.260	85.2
0.015	0.532	78.2	0.286	95.3
